# The Psychometric Assessment of the HIV/AIDS Questionnaire in the Multiple-Indicator Cluster Survey (MICS-6)

**Published:** 2019-05

**Authors:** Mohtasham GHAFFARI, Yadollah MEHRABI, Sakineh RAKHSHANDEROU, Ardeshir KHOSRAVI, Hooman BAKHSHANDEH, Narjes TAVAKOLIKIA, Elham ELAHI

**Affiliations:** 1. Department of Public Health, Environmental and Occupational Hazards Control Research Center, School of Public Health and Safety, Shahid Beheshti University of Medical Sciences, Tehran, Iran; 2. Department of Epidemiology, School of Public Health and Safety, Shahid Beheshti University of Medical Sciences, Tehran, Iran; 3. Deputy for Public Health, Ministry of Health and Medical Education, Tehran, Iran; 4. Rajaie Cardiovascular Medical and Research Center, Iran University of Medical Sciences, Tehran, Iran; 5. Department of Culture, Tehran University of Medical Sciences, Tehran, Iran; 6. Department of Health Education and Health Promotion, School of Public Health and Safety, Shahid Beheshti University of Medical Sciences, Tehran, Iran

**Keywords:** Psychometrics, HIV/AIDS, Multiple-indicator cluster survey-6

## Abstract

**Background:**

This study aimed to psychometrically assess the HIV/AIDS items of MICS-6 in the Iranian population in the effort to collect valid and reliable data regarding the Iranian culture.

**Methods:**

This methodological study is a psychometric assessment of the HIV/AIDS items in the Multiple-Indicator Cluster Survey (MICS) as the AIDS module in MICS Round 6, which includes items on awareness and stigma in people aged over 15 and examines them for their HIV test history. First, the AIDS module was translated into Persian and then translated back into English; subsequently, the psychometric properties of the Persian version were assessed. The face, content and construct validities of this version were also evaluated. The test-retest and internal consistency estimates were also used to determine the instrument’s reliability, and the questionnaire was implemented in 200 samples from the target population as a pilot study.

**Results:**

The validity of the instrument was confirmed with a CVR higher than 0.78 and a CVI of 0.79. The exploratory factor analysis was performed to confirm the validity of the five factors of the MICS-6. A high internal consistency was reported with a Cronbach’s alpha coefficient of 0.98 for the questionnaire as a whole and questionnaire has good validity and reliability.

**Conclusion:**

The Persian version of the AIDS module of the MICS-6 has satisfactory reliability and validity. The present findings are consistent with the results of other studies on the psychometrics of the international AIDS questionnaire.

## Introduction

HIV/AIDS is among the major challenges of the modern era. According to statistics published at the end of 2017, 36.9 million people have been infected with this virus and 940000 have died due to AIDS-related causes in 2017 (1). According to a report by the Infection Disease Management Center of the Ministry of Health and Medical Education of the Islamic Republic of Iran, 37650 HIV-positive people have been identified, registered and reported until the end of Feb 2018, including 83% men and 17% women. About, 51% of the patients are in the 21 to 35 age group (2). To emphasize the importance of HIV/AIDS, the sixth goal of the Third-Millennium Development Goals (MDGs) is to prevent the spread of AIDS (3). Moreover, goal 3.3 of the 17 Sustainable Development Goals (SDGs) is to eliminate AIDS epidemics by 2030 (new HIV infections per 1000 non-infected people based on gender, age and key populations) (4). At the national level, the HIV/AIDS control section of the program for reducing high-risk behaviors and HIV as part of the Health Reform Plan started in 2015 and reveals the importance of addressing this disease in long-term policymaking (5).

At present, promoting public awareness and improving attitudes (in the 15–49 age group) with regard to the prevention of AIDS and increasing the percentage of young men and women who are fully aware of the routes of HIV transmission and do not accept any misconceptions about AIDS/HIV prevention are part of the national commitments and priorities of the AIDS programs established by the Ministry of Health and Medical Education (6).

In response to the weaknesses existing in the current health information systems of countries, and in particular third-world countries, standard surveys have been designed, and the Multiple-Indicator Cluster Survey (MICS) is among these tools. The UN Children’s Fund has implemented the MICS since 1996 in six rounds and in more than 60 countries. Iran’s Multiple Indicator Demographic and Health Survey (IrMIDHS) has been carried out in Iran in 2010 and 2015 using the full scientific and executive capacity and potential of the country. The major indicators of the MDGs, regarded as the country’s international obligations, were prepared and reported based on these studies (7,8). IrMIDHS translate and use the exact international questionnaire regardless of the cultural differences in place, but this procedure is not specific to surveys performed in Iran, as nearly most of the countries carrying out this survey merely translate the main items of the questionnaire to their official language and then translate it back into its original language (9,10). The revalidation of this instrument is necessary before it can be used in a culture other than the cultures for which it has been developed (11) since IrMIDHS is part of the MAP of Iranian National Health Surveys and is recommended to be re-implemented every five years (12).

It is therefore essential to methodologically study the HIV/AIDS-related module of the IrMIDHS (containing items on awareness and stigma) in people aged 15 and over based on the Iranian culture. The present article showed the process of localization and psychometric assessment of this instrument for the measurement of DHS.

## Materials and Methods

The data collection tool used in this study was MICS-6. The English version of the questionnaire consists of 36 items about AIDS and has been developed to assess three areas, including awareness, stigma and the HIV test history throughout life. The items are answered with ‘yes’, ‘no’, or ‘I do not know’. MICS-6 is interpreted using the instructions given in relevant sources (13). The AIDS module studies women and men over age 15 separately by gender, and the difference between the two forms of the questionnaire is that the female form has pregnancy-related items examining the last two years.

### Translation Validation

First, the AIDS module of MICS-6 was translated into Persian. After the confirmation of the initial translation by the research team, the Persian questionnaire was distributed among an English-speaking person for re-translation into English. After receiving the English translation of the questionnaire, the two English versions were compared and the preliminary mistakes in translation and retranslation were resolved and the final Persian version was prepared for submission to experts and for the other psychometric steps.

### Face Validity

Quantitative and qualitative methods were used to determine the face validity of this questionnaire (14). For the qualitative assessment, the opinions of 15 experts in fields of health, epidemiology, social sciences, AIDS Fellowship, reproductive health, infectious diseases and social medicine were obtained on the questionnaire’s level of difficulty and the irrelevance and ambiguity of its items through individual interviews. For the quantitative assessment, 30 individuals over age 15 were asked to examine each of the items with regard to their significance based on a 5-point Likert scale. The impact score of the items was then calculated based on the formula. All the items with an impact score above 1.5 were kept for the subsequent analysis.

### Content Validity

To determine the CVR, the questionnaire was sent to 15 experts asked to examine and score each item based on a three-part scale (ranging from ‘necessary’ to ‘unnecessary’). The CVR was then calculated based on the formula and its numerical value was determined using Lawshe’s table (15).

Regarding the CVI, four criteria, including simplicity, conciseness, relevance and clarity, were separately examined for the items based on a four-point Likert scale and CVI was determined using the relevant formula. The items with a score higher than 0.79 were accepted (15).

### Construct Validity

The Exploratory Factor Analysis was used to determine the construct validity of the items (16). Factor analysis was performed using the Principle Component Analysis (PCA) method adjusted for nominal variables. Equamax and Kaiser Normalization were used for rotation.

### Instrument Reliability

The test-retest and internal consistency methods were used to assess the reliability of the tool (17). The test was implemented two times (at an interval of about two weeks) in 30 subjects (who were not the same as the main sample population) and their scores were compared. The test reliability coefficient, i.e. the correlation coefficient between the scores obtained in the test and the retest (18), was considered acceptable if above 0.7 and satisfactory if between 0.85 and 0.95 (19). Cronbach’s alpha coefficient was used to determine the internal consistency of each subscale and the entire questionnaire. Cronbach’s alpha represents the relevance of a group of items that measure a construct and specifies that the scale most likely measures a coordinated variable (20). A minimum Cronbach alpha of 0.7 is desirable for exploratory studies, but if the tool is to have good internal validity, this coefficient must be at least 0.7 to 0.8 (21).

### Study Population

The pilot stage of this methodological study was carried out in a population of people from both genders, aged over age 15, residing in Tehran and from different age and education backgrounds. Ten samples were taken per each item for the exploratory factor analysis (20), and a sample size of 180 was therefore studied based on the number of the items in the questionnaire. To take account of sample loss, 200 people were ultimately entered into the study. Personal consent was obtained from the participants and they could withdraw at any stage if they were no longer willing to continue the study.

### Ethical aspects

The participants were briefed on the study objectives and methods in a clear and concise manner. They were free to participate or not participate in the research. Informed consent was obtained from the participants before beginning the study. Moreover, this project has been approved by the Ethics Committee of the National Institute of Health Research under the code IR.TUMS.NIHR.REC.1396.44.

## Results

The CVR was above 0.78 for all the items, and the content validity of the items was thus approved. The CVI of the questionnaire was reported as 0.79. The validity of the study tool was therefore approved. The item “Is it possible for people to be infected with HIV by magic or supernatural causes?” obtained a CVI less than 0.79 and was therefore removed.

The number of factors in the factor analysis was taken based on Eigen values ≥1. The results of the model after rotation showed that five factors can be proposed for this questionnaire, which account for 77.4% of the total variance. The loading value was measured after rotation for the five proposed factors using factor analysis. The factor loading was set at 0.4 for all the factors.

The following headlines can be given to the items based on their content: Factor 1: Sexual Transmission; Factor 2: Non-sexual transmission; Factor 3: Prevention; Factor 4: Test history; Factor 5: Stigma.

This categorization is consistent with the main axes of the questionnaire. The constructs found in this analysis, therefore, suggest that the structure of the questionnaire is appropriate for achieving the expected goals (Table 1). Regarding the compatibility of the questionnaire, the obtained factors and the items placed in each factor are in accordance with the mentality and design framework of the main MICS. These findings thus confirm the reliability and validity of the questionnaire.

**Table 1:** Results of factors analysis

***Question***	***Code***	***Factor 1***	***Factor 2***	***Factor 3***	***Factor 4***	***Factor 5***
HA2	Can people reduce their chance of getting HIV by having just one uninfected sex partner who has no other sex partners?	.843				
HA4	Can people reduce their chance of getting HIV by using a condom every time they have sex?	.917				
HA3	Can people get HIV from mosquito bites?		.700			
HA5	Can people get HIV by sharing food with a person who has HIV?		.663			
HA7	Is it possible for a healthy-looking person to have HIV?		.818			
HA8	CAN HIV BE TRANSMITTED FROM A MOTHER TO HER BABY:					
	(A) During pregnancy?					
	(B) During delivery?					
	(C) By breastfeeding?	.918				
HA10	Are there any special drugs that a doctor or a nurse can give to a woman infected with HIV to reduce the risk of transmission to the baby?			.533		
HA13	During any of the antenatal visits for your The last pregnancy, were you given any information about:					
	(A) Babies getting HIV from their mother?					
	(B) Things that you can do to prevent getting HIV?					
	(C) Getting tested for HIV?					
	(D) Were you Offered a test for HIV?		.769			
HA14	I don’t want to know the results, but were you tested for HIV as part of your antenatal care?				.675	
HA15	I don’t want to know the results, but did you get the results of the test?				.952	
HA16	After you received the result, were you given any health information or counseling related to HIV?				.818	
HA18	Between the time you went for delivery but before the baby were born were you offered an HIV test?				.918	
HA19	I don’t want to know the results, but were you tested for HIV at that time?				.602	
HA20	I don’t want to know the results, but did you get the results of the test?				.923	
HA22	Have you been tested for HIV since that time you were tested during your pregnancy?				.880	
HA23	How many months ago was your most recent HIV test?				.602	
HA30	Would you buy fresh vegetables from a shopkeeper or vendor if you knew that this person had HIV?					.880
HA31	Do you think children living with HIV should be allowed to attend school with children who do not have HIV?					.771
HA32	Do you think people are reluctant to do HIV testing because they are afraid of responding to others who are likely to respond positively?					.896
HA33	Do people talk badly about people living with HIV, or who are thought to be living with HIV?					.770
HA34	Do people living with HIV, or thought to be living with HIV, lose the respect of other people?					.610
HA35	Do you agree or disagree with the following statement?					
	I would be ashamed if someone in my family had HIV.				.782	
HA36	Do you fear that you could get HIV if you come into contact with the saliva of a person living with HIV?					.850

After modifying the questionnaire, its internal consistency and Cronbach’s alpha coefficient were also calculated to determine its reliability. Cronbach’s alpha was 0.98 for the questionnaire as a whole (Table 2). Moreover, the ICC of 0.98 was deemed appropriate. The questionnaire was completed in the 30 participants after an interval of two weeks, and the scores were compared between the two runs. The insignificant differences in the scores of the questionnaire between the two runs indicate the repeatability of the tool (*P*=0.744) (Table 2).

**Table 2:** Internal consistency based on analysis

***Dimension***	***Cronbach’s Alpha***	***% of Variance***
1	.720	18.725
2	.697	16.836
3	.726	16.680
4	.616	13.213
5	.581	12.019
Total	.981	77.473

In the pilot study, all the 200 samples participated in the pilot psychometric study, including 104 female (52%) and 96 male (48%) samples, 141 married (70.5%) and 54 single samples (27%) and five widows/widowers (2.5%). The mean age of the subjects was 46.5 yr with a standard deviation of 15. Totally, 108 were employed (54%), 29 (14.5%) had incomes without a job, 29 (14.5%) were housewife, 24 (12%) were students and the rest did not fit into any of the categories.

## Discussion

The present study was a psychometric assessment of the HIV/AIDS items of MICS-6 in the Iranian population and aimed to offer a standard, native and reliable questionnaire using the views of the target group and an expert panel and has used scientific psychometric methods to determine the reliability of the questionnaire. No articles were obtained on the psychometrics of the AIDS module of the MICS-6 in Iran or other countries with which to compare the present findings. In Iran, most research on AIDS-related awareness and stigma has been conducted with researcher-made tools and it is not possible to discuss them in this study due to their non-compliance with the required standards; for instance, the target group has been ignored in some surveys, the data provided in most of the studies are ambiguous and inadequate and the number of participants in the expert panel is considered low (22). Nevertheless, our results showed that the Persian version of the AIDS module of MICS-6 has a desirable validity, which is in line with the results of other studies conducted for the psychometric assessment of the international AIDS questionnaire (23–25). The reliability testing showed that all five factors and the questionnaire as a whole have acceptable internal consistency coefficients, which is consistent with the results of other studies (23–25). The test-retest results showed that the questionnaire has high retest reliability, which indicates that the items are not affected by external factors and the scores are stable over time. Similar results have been reported (23–25).

Since most studies on the subject have not used a standard tool and have failed to assess the various dimensions of awareness and stigma regarding AIDS and have yielded incorrect data, it is impossible to make a cross-cultural comparison of the subject. The need for a native tool to measure public awareness and attitudes (in the 15–49 age group) regarding the prevention of AIDS is more pressing than before, and AIDS policy-makers should target the promotion of awareness and the elimination of stigma on this subject in different areas. The present study was therefore designed for the psychometric assessment of the AIDS module of MICS in Iran. This questionnaire is a standardized tool for assessing the dimensions of awareness and attitude toward HIV infection with factors including sexual transmission, non-sexual transmission, prevention, test history and stigma, and if localized, can be used as an appropriate tool in different cultures and communities. The limitations of this research include the difficulty of access to male samples for completing the questionnaire.

## Conclusion

The Persian version of the AIDS module of MICS-6 has appropriate reliability and validity. Use of present questionnaire with approved validity and reliability helps us to avoid re-perform a national study with heavy costs imposed on the health system and health policy-makers and facilitates the collection of accurate national data that can be compared with global data. Other modules of MICS-6 are recommended to also be psychometrically assessed and validated for applying in Iranian population.

## Ethical considerations

Ethical issues (Including plagiarism, informed consent, misconduct, data fabrication and/or falsification, double publication and/or submission, redundancy, etc.) have been completely observed by the authors.

## References

[1] World Health Organization. Key facts of HIV/AIDS, (2018). http://www.who.int/mediacentre/factsheets/fs360/en/

[2] Ministry of Health HIV / AIDS statistics. Tehran, Iran: Ministry of Health, Center for Disease Management (2018).(In Persian)

[3] World Health Organization (2014). Millennium Development Goals. https://www.who.int/topics/millennium_development_goals/diseases/en/

[4] World Health Organization (2015). UN Sustainable Development Goals. https://sustainabledevelopment.un.org/?menu=1300

[5] Moradi-Lakeh M Vosoogh-Moghaddam A (2015). Health Sector Evolution Plan in Iran; Equity and Sustainability Concerns. Int J Health Policy Manag, 4(10):637–40 26673172 10.15171/ijhpm.2015.160 PMC4594102

[6] Ministry of Health and Medical Education Aids diseases in IRAN and world- (2001)

[7] Hossinpour A Mohammad K Majdzadeh R (2005). Socioeconomic inequality in infant mortality in Iran and across its provinces, Bull World Health Organ, 83(11):837–44. 16302040 PMC2626462

[8] Rashidian A Khosravi A Arab M (2011). IrMIDHS-I.R. Iran. Multiple-Indicator and Demographic Health Survey 2010: Questionnaires, guides and protocols. Tehran: National Institute of Health Research and Deputy for Health, Ministry of Health and Medical Education. (In Persian).

[9] Ministry of Health and Population, Cairo, Egypt El-Zanaty and Associates Cairo, Egypt. The DHS Program, ICF International, Rockville, Maryland, USA 5 2015. Egypt Demographic and Health Survey 2014. (Accessed 2016)

[10] Kenya National Bureau of Statistics, Nairobi, Kenya. Ministry of Health, Nairobi, Kenya. National AIDS Control Council, Nairobi, Kenya. Kenya Medical Research Institute, Nairobi, Kenya. National Council for Population and Development Nairobi, Kenya The DHS Program, ICF International, Rockville, Maryland, USA. Kenya Demographic and Health Survey 2014. (Accessed 2016)

[11] Krishna VAS Bhatt RS Chandra PS (2005). Unheard voices: experiences of families living with HIV/AIDS in India. Contemp Fam Ther, 27(4): 483–506.

[12] Rashidian A Khosravi A Khabiri R (2014). Final report: Designing A Master Plan for Household Survey in Iran. (in Persian)

[13] Multiple Indicator Cluster Surveys. 2018 http://mics.unicef.org/tools

[14] DeVon HA Block ME Moyle-Wright P (2007). A psychometric toolbox for testing validity and reliability. J Nurs Scholarsh, 39(2): 155–164. 17535316 10.1111/j.1547-5069.2007.00161.x

[15] Broder HL McGrath C Cisneros GJ (2007). Questionnaire development: face validity and item impact testing of the Child Oral Health Impact Profile. Community Dent Oral Epidemiol, 35 Suppl 1:8–19. 17615046 10.1111/j.1600-0528.2007.00401.x

[16] Ellenbecker CH Byleckie JJ (2005). Home health care nurses’ job satisfaction scale: Refinement and psychometric testing. J Adv Nurs, 52(1):70–8. 16149983 10.1111/j.1365-2648.2005.03556.x

[17] Sun TK Chiu SC Yeh SH Chang KC (2006). Assessing reliability and validity of the Chinese version of the stroke scale: scale development. Int J Nurs Stud, 43(4): 457–463. 16146632 10.1016/j.ijnurstu.2005.07.004

[18] Saif AA (2013). Educational Measurement, Assessment and Evaluation. Tehran Nashre Douran. (In Persian)

[19] Speziale HS Streubert HJ Carpenter DR (2011). Qualitative research in nursing: Advancing the humanistic imperative. Lippincott Williams & Wilkins.

[20] Nouri Parkestani H Alimohammadi I Arghami S (2010). Assessment of reliability and validity of a new safety culture questionnaire. Iran Occupational Health, 7(1):18–25. (In Persian)

[21] Kuo CL Turton MA Lee-Hsieh J (2007). Measuring peer caring behaviors of nursing students: Scale development. Int J Nurs Stud, 44(1): 105–114. 17027986 10.1016/j.ijnurstu.2006.07.025

[22] Vakili M Babakhani L Sharifi S (2018). Development and Psychometric Analysis of A 44-Item HIV/AIDS Knowledge Scale: An Iranian Cultural and Population Based Study. Iran J Epidemiol, 14 (2):116–125. (In Persian)

[23] Davis C Sloan M Macmaster S (2007). The International AIDS Questionnaire-English Version (IAQ-E). Journal of HIV/AIDS Prevention in Children & Youth, 7(2):29–42.

[24] Razavi P Hajifathalian K Saeidi B (2012). Quality of Life among Persons with HIV/AIDS in Iran: Internal Reliability and Validity of an International Instrument and Associated Factors. AIDS Res Treat, 2012:849406. 22292116 10.1155/2012/849406 PMC3265053

[25] Eskandari N Alipour Z Lamyian M (2013). Validity and reliability of the international AIDS questionnaire for Iranian student population. Arak Medical University Journal, 15(69): 1–12.

